# Endoscopic Removal of Intrauterine Contraceptive Device Perforating Into the Bladder: A Case Report and Review of Literature

**DOI:** 10.7759/cureus.35839

**Published:** 2023-03-06

**Authors:** Amr H Wahba, Maximilian Mattes Auer-Schmidt, Torsten Schmidt

**Affiliations:** 1 Department of Obstetrics and Gynecology, Cairo University Hospital, Cairo, EGY; 2 Department of Gynecologic Endoscopy, PAN-Klinik, Cologne, DEU; 3 Department of Obstetrics and Gynecology, Cologne University Hospital, Cologne, DEU

**Keywords:** urinary bladder stone, bladder perforation, uterine perforation, hysteroscopy, intrauterine contraceptive devices

## Abstract

Intrauterine contraceptive device (IUCD) is a commonly used contraceptive method with the advantage of being a long-acting and reversible contraceptive method. However, its insertion can be rarely associated with serious complications such as uterine perforation, which can more rarely result in injury of the nearby viscus. In this report, we document a rare case of IUCD perforation of the uterus and bladder, its diagnosis using transvaginal ultrasonography and hysteroscopy, and management using a minimally invasive approach with a satisfactory patient outcome.

## Introduction

Intrauterine contraceptive device (IUCD) is the most common method used for reversible contraception in women because it is safe, long-acting, and cost-effective [1]. One of the most serious complications of IUCD is perforation of the uterus with subsequent migration of the device into pelvic or abdominal organs. The overall reported incidence of intrauterine device perforation is about 0.87 per 1,000 insertions [2]. It has been reported with all types of devices including Lippes loop, Dalkon Shield, copper IUCDs, the intrauterine system (IUS), and GyneFix [3].

Perforation of the IUCD through the uterine wall into the bladder is even rarer and has been reported as case reports with at least 40 cases reported in the literature [4]. It can be a complete perforation, where the IUCD floats freely in the urinary bladder, or partial perforation. Such cases were thought to cause fistula formation, and thus open approach with cystotomy was the standard surgical management to remove the IUCD and repair the fistulous tract surgically. This case report describes a minimally invasive approach using the hysteroscope to perform cystoscopy for trans-urethral removal of perforating IUCD from within the bladder wall without subsequent fistula formation.

## Case presentation

A 28-year-old G1P1L1 patient presented to the outpatient clinic of the Department of Obstetrics and Gynecology at Cairo University Hospital for removal of an IUCD 8 months after its insertion, which had taken place at an external family-planning facility. The IUCD was inserted 40 days following normal vaginal delivery. Shortly after the insertion, the patient started to be troubled with persistent symptoms of urinary tract infection (UTI) in the form of dysuria, frequency, and nocturia that did not resolve completely despite repeated courses of antibiotics. Her urine analysis revealed the presence of inflammation showing significant pyuria with microscopic hematuria with the absence of crystals. Urine culture was negative. She was advised to remove the IUCD. Following a failed attempt at removal at an external outpatient facility, she was referred to our hospital. 

**Figure 1:**
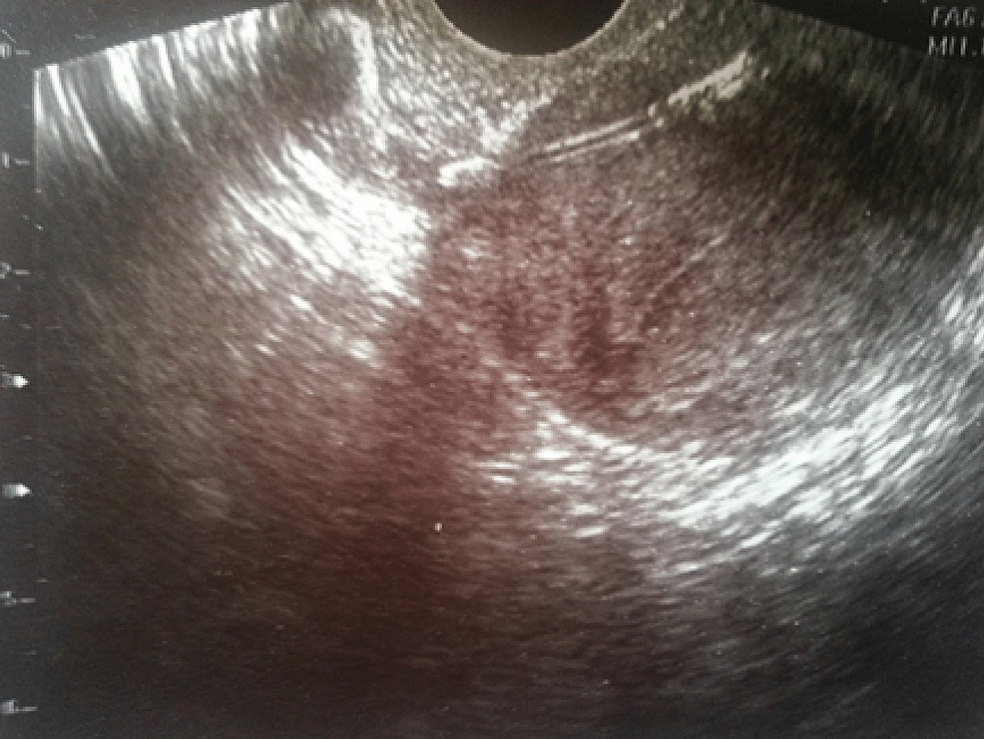
TVS with empty bladder showed IUCD to be embedded through the myometrium of the anterior uterine wall TVS: transvaginal ultrasound; IUCD: intrauterine contraceptive device

Initial assessment with transvaginal ultrasound (TVS) (with empty bladder) showed the IUCD to be embedded in the anterior wall of the uterus (Figure 1). So the patient was referred to our outpatient hysteroscopy unit for assessment.

**Figure 2:**
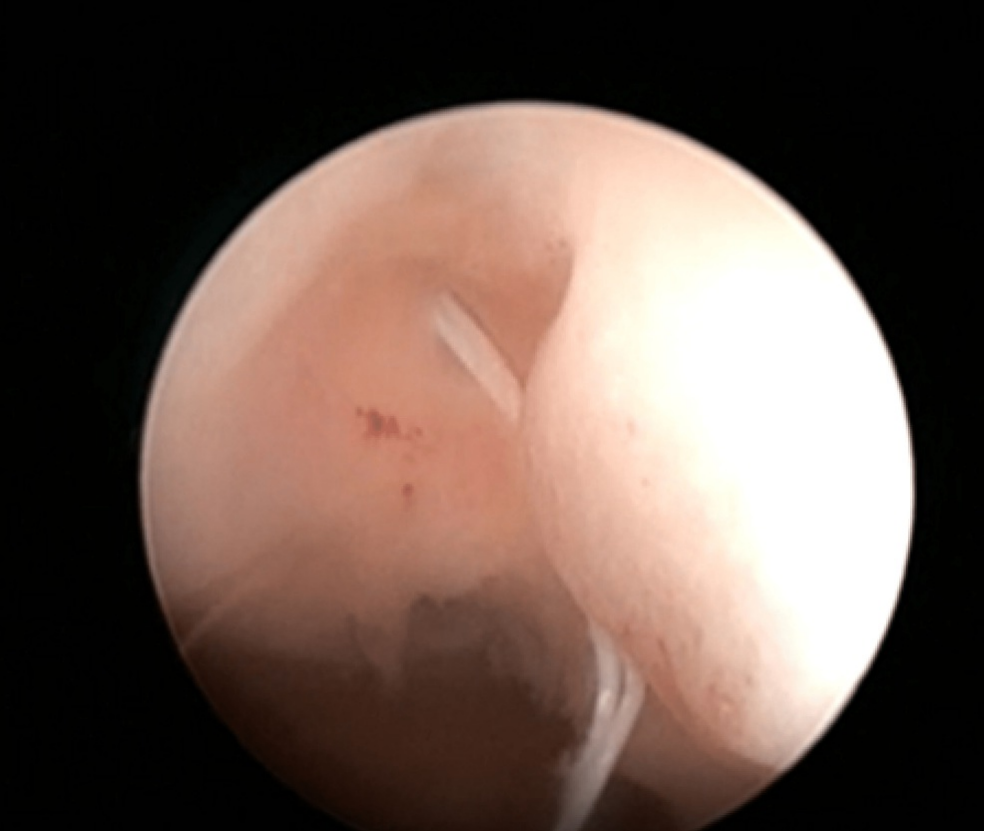
Outpatient hysteroscopy showing threads of IUCD seen inside the uterine cavity perforating through the anterior wall IUCD: intrauterine contraceptive device

Outpatient diagnostic hysteroscopy revealed threads of IUCD inside the uterine cavity passing through the anterior uterine wall (Figure 2). Although tempting to pull on the threads, the procedure was aborted for further evaluation.

**Figure 3:**
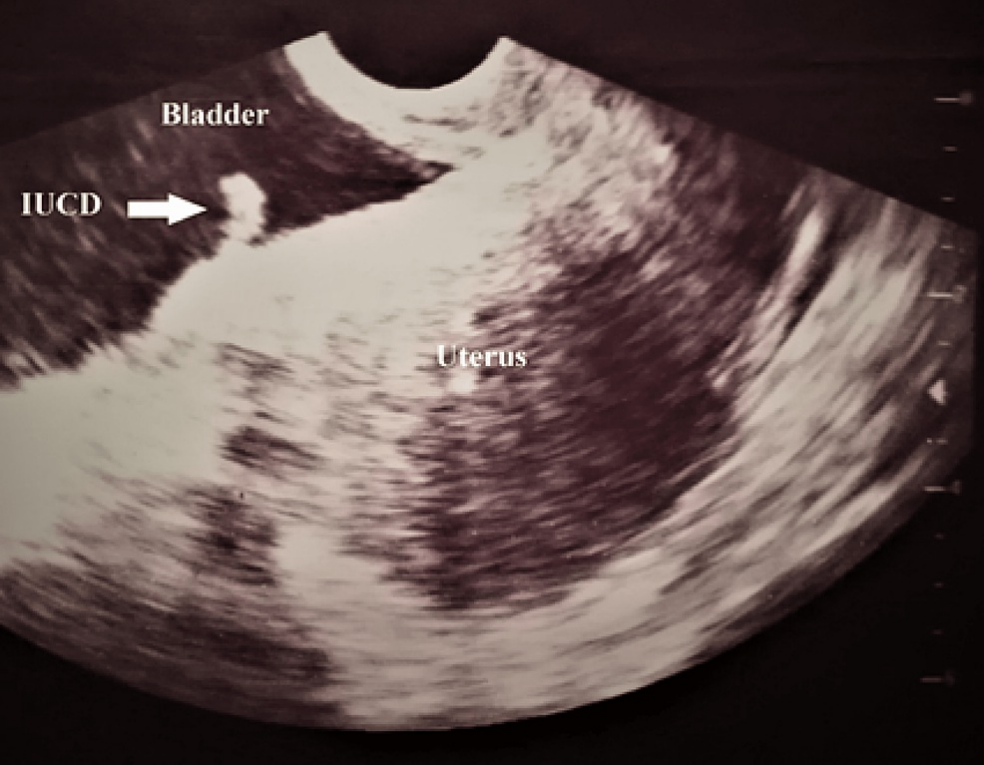
TVS with full bladder showing the IUCD (arrow) within the bladder cavity jutting from the posterior wall of the bladder TVS: transvaginal ultrasound; IUCD: intrauterine contraceptive device

TVS was repeated with a full bladder, which at that time showed the IUCD penetrating through the posterior bladder wall (Figure 3).

**Figure 4:**
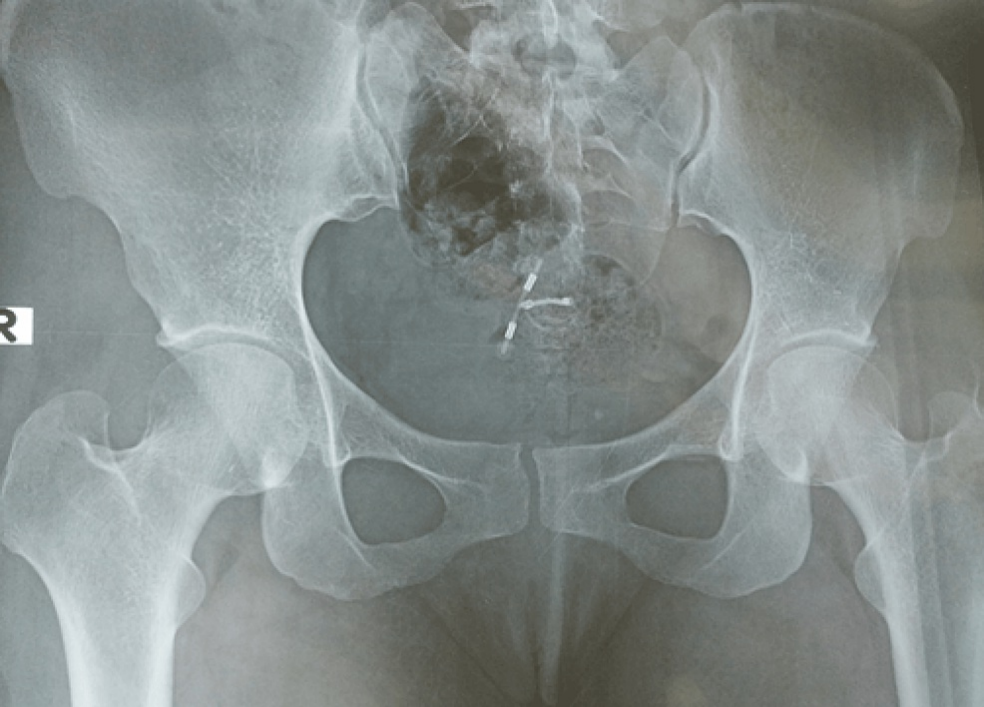
Plain AP X-ray showing IUCD in the pelvis but with malposition (note the halo around the lower arm of the IUCD representing shadow from the urinary stone) AP: anteroposterior; IUCD: intrauterine contraceptive device

A plain X-ray pelvis was done and revealed malposition of the IUCD with the presence of a halo around the tip of one of the IUCD arms. This finding indicates a urinary stone formation on top of the IUCD (Figure 4).

**Figure 5:**
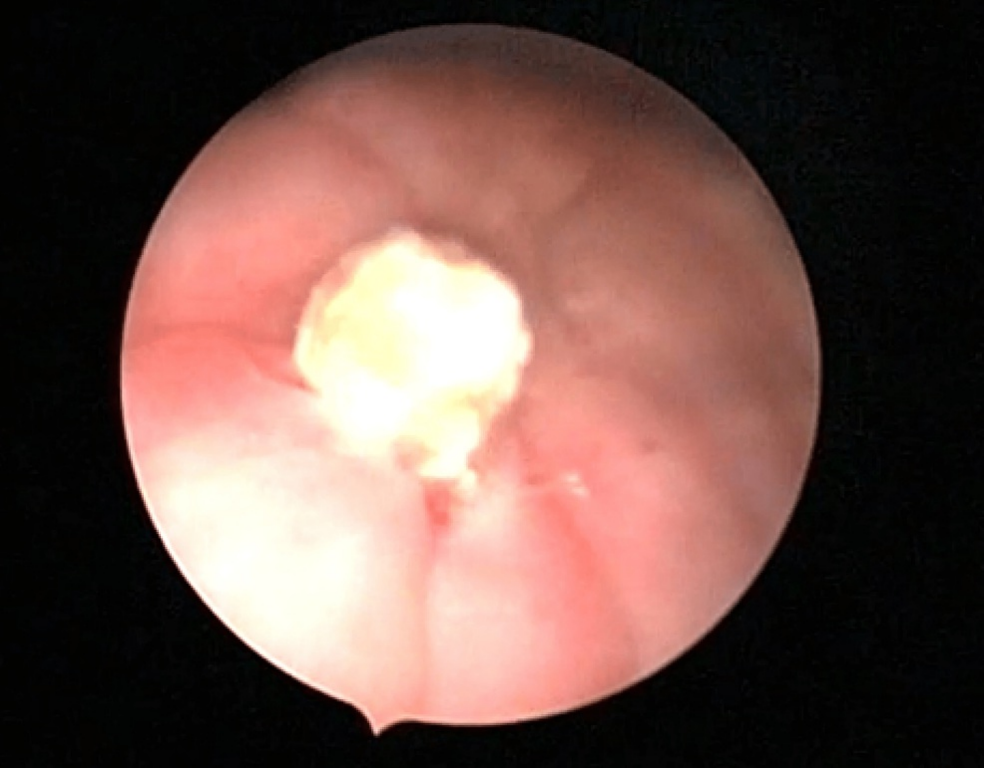
Cystoscopy showing stone formed on top of the perforating arm of the IUCD IUCD: intrauterine contraceptive device

We scheduled the patient post-menstrually for hysteroscopy and cystoscopy under general anesthesia. We performed cystoscopy using a 2.7-mm rigid continuous-flow hysteroscope with a 30-degree angle, an outer diameter of 5 mm, and a 5-French working channel. Cystoscopy showed a spherical hard stone about 1.5 x 1 cm formed on top of the transverse limb of the IUCD which was perforating through the posterior wall of the bladder (Figure 5).

**Figure 6:**
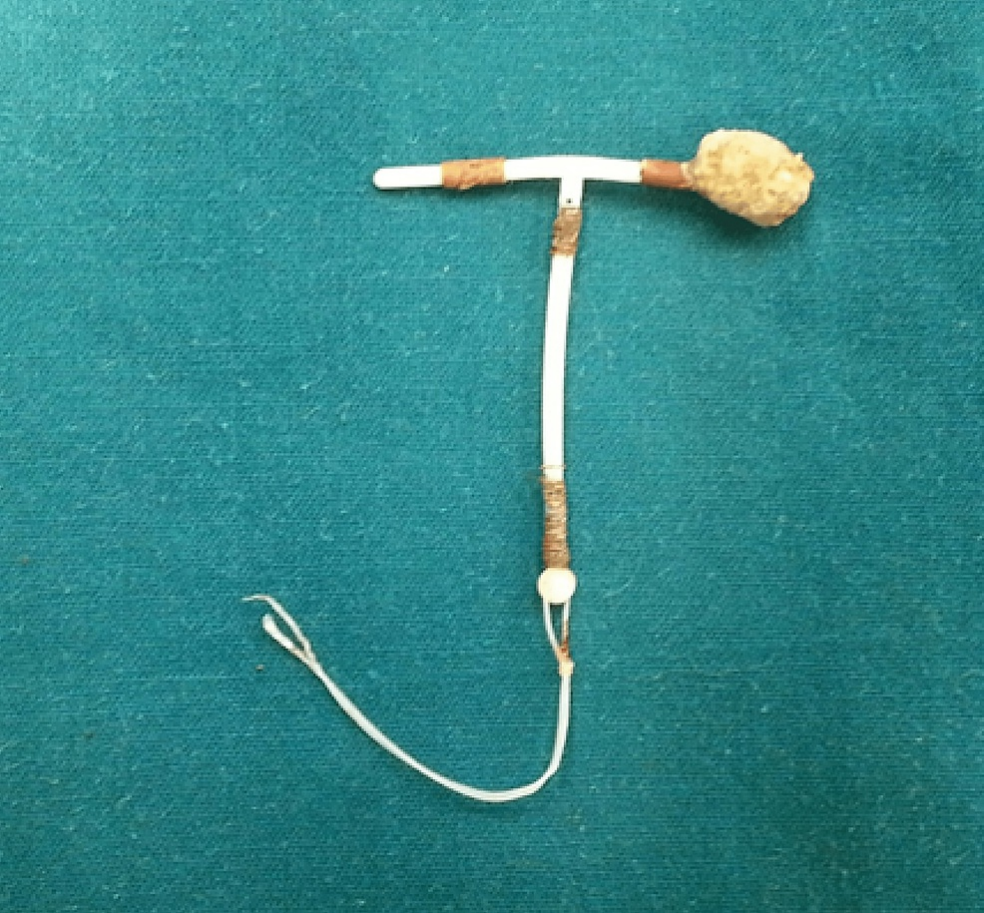
Copper IUCD with stone formed at the tip of the intravesical arm Intrauterine contraceptive device

An attempt to pull on the IUCD by grasping the stone, failed as the stone did not fragment and was slippery. Thus, the arm of the IUCD was firmly held by a grasper just below the stone and pulled out bringing the whole IUCD inside the bladder, which was then removed via per urethral route by pulling on the threads (Video 1). Figure 6 shows the copper IUCD with a hard spherical stone formed around the intravesical arm after extraction. An indwelling catheter was inserted at the end of the operation.

**Video 1:** Video demonstrating diagnosis and management of IUCD perforating the bladder IUCD: intrauterine contraceptive device

The patient had an uneventful postoperative recovery. The catheter was removed after a few hours postoperatively and the patient voided normally. She was discharged later the same day. Follow-up was arranged after 1 week, 1 month, and 3 months and did not reveal any postoperative complications or fistulous formation with complete resolution of the patient's urinary symptoms. The patient’s consent for the procedure and for the publication of the case report was obtained.

## Discussion

Uterine perforation is a potentially serious complication of IUCD insertion that can result in loss of the contraceptive effect, weakening of the uterine wall with a risk of rupture in subsequent pregnancies, and injury of the viscera with a theoretical risk of fistula formation.

It is thought that uterine perforation may be primary at the time of insertion; undetected extreme retroverted uterine position is the most common reason for perforation [5]. Secondary perforation of the uterus can occur by slow migration of the IUCD, which is thought to be augmented by spontaneous uterine contraction and bladder contractions [6]. In our case, we believe that perforation occurred at the time of insertion since symptoms developed shortly after the insertion of the IUCD. 

Uterine perforation by IUCD may remain silent and pass unnoticed. Some cases are not identified until months or years after insertion [7]. However, most patients with intravesical migration of IUCD are symptomatic with UTI being the most common presentation (8). The patient in this case presented with persistent UTI. Once an IUCD has penetrated the bladder, it usually becomes encrusted with calculi [8].

A careful ultrasound examination is usually the first line of investigation and will establish the diagnosis in most cases. When the IUCD is seen embedded into the anterior uterine wall, it is important to re-do the TVS with a full bladder as this will provide a more accurate diagnosis of bladder perforation. A plain X-ray of the pelvis will confirm the diagnosis of lost IUCD and, more importantly, will help to identify the presence of urinary stones formed on top of the IUCD. 

A literature review revealed varied treatment options for an IUCD perforating the bladder. IUCDs that are located completely inside the bladder (complete perforation), where the IUCD is floating with or without stone formation, can be easily removed via per urethral route using cystoscopy without any risks [9]. Large stone formations over the IUCD can be fragmented by lithotripsy to facilitate its cystoscopic retrieval. An open approach with cystotomy should only be considered in cases of large stones over IUCD that are difficult to be managed by the endoscopic route [6].

For IUCDs with partial penetration of the bladder wall, open surgery has been used generally as the standard treatment in order not only to remove the device but also to repair the defect to decrease the theoretical risk of fistula formation [10]. However, this type of open surgery increases morbidity because of extensive surgical exploration and prolonged hospital stay. Laparoscopic removal, a minimally invasive alternative to open surgery, has been described with less morbidity than open surgery [11]. However, cystoscopic removal, as done in our case, represents the least invasive procedure and has been described successfully in recent case reports [4,5, 6, 12]. 

Per vaginal removal may represent another minimally invasive approach for cases with IUCD partial perforation of the bladder, provided that the threads of IUCD are accessible vaginally and at the same time there is no stone formation on the vesical part of the IUCD. Kiilholma et al., 1989, retrieved IUCD vaginally by pulling on the threads of the IUCD whose horizontal arm perforated through the bladder wall without stone formation. A catheter was placed for 5 days [13]. A similar case was also reported by Elleithy et al., 2008, who left an indwelling catheter for 2 days [14]. Both cases were uncomplicated with no fistula.

In our case, the threads of the IUCD were visible inside the uterine cavity with the use of hysteroscopy; however, we avoided hysteroscopic removal by pulling on the IUCD threads, although temptingly feasible, to avoid possible widening of the fistulous tract by the stone formed on the vesical part of the IUCD, which we believe could have increased risk of fistula formation.

Although the cystoscopic approach and per vaginal approach allowed retrieval of IUCD partially embedded in the bladder wall without closure of the defect, paradoxically, no fistula formation has been reported as a complication so far.

It may be thought that the use of an indwelling catheter with prolonged bladder drainage postoperatively may have contributed to the decreased risk of fistula formation in these cases [5]. However, our case report questions this assumption, as the catheter was removed the same day and the patient did not develop a fistula. On the other hand, prolonged bladder drainage may not be protective of vesicouterine fistula, as evident from the single report of menouria developed due to a vesicouterine fistula following removal of perforated IUCD despite bladder drainage for 2 months [15]. Table 1 shows the duration of bladder drainage in cases with partial bladder perforation managed with a minimally invasive approach.

**Table 1:** Postoperative bladder drainage following minimally invasive approach for removal of IUCD partially perforating the bladder IUCD: intrauterine contraceptive device

Case report	Cases with partial perforation	Approach	Duration of postoperative bladder drainage	Discharge
Sallami et al., 2011 [5]	9 cases	Cystoscopy	10 days	Not stated
Sawant et al., 2015 [6]	2 cases	Cystoscopy	7 days in one case, 14 days in other case	After 3-4 days of surgery
Kart et al., 2015 [12]	1 case	Cystoscopy	1 day	After 1 day
Dimitropoulos et al.,2016 [4]	1 case	Cystoscopy	Duration Not stated	3rd postoperative day
Ko et al., 2011 [16]	1 case	Cystoscopy	14 days	Not stated
Kiilhoma et al., 1989 [13]	1 case	Vaginal approach	5 days	Not stated
Elleithy et al., 2008 [14]	1 case	Vaginal approach	2 days	Not stated

In this case, the early removal of Foley’s catheter improved the patient’s satisfaction, hastened her recovery, and shortened her hospital stay - a favorable outcome for a patient who had sustained a previous complication with IUCD insertion.

We believe that the non-occurrence of a fistula despite the non-closure of the defect can be explained by different factors. The muscular wall of the uterus may enhance the closure of the tract by contraction of the uterine muscle after retrieval of the IUCD. The inherent tone of the myometrium may also contribute to higher intrauterine pressure compared to the intravesical pressure preventing the flow of urine from the bladder to the uterus. The oblique course of the IUCD may also play an important protective mechanism. The small size of the defect left after extraction of IUCD may be within the capacity of the bladder to seal. Scheduling the procedure postmenstrual allows time for the healing of the defect before the next period.

Since the occurrence of such a condition is quite rare, evidence for the diagnosis and management of IUCD perforating into the bladder will rely only on case reports. The case reported here showed the ability to diagnose the condition using TVS with a full bladder and supports the use of the cystoscopic approach for the removal of partial IUCD perforation of the bladder. Early catheter removal and same-day discharge significantly improved patient satisfaction, with no fistula formation. Thus, prolonged post-operative bladder drainage may not be required. More evidence to support this supposition is needed through future case reports.

## Conclusions

Persistent urinary symptoms should provoke careful assessment of the IUCD location. TVS with a full bladder is helpful to establish the diagnosis of IUCD perforating into the bladder. Improving the satisfaction of patients who have sustained a previous complication is very important and can be achieved by using the least invasive approach and shortening hospital stay. Cystoscopic retrieval of an IUCD partially perforating into the bladder is safe and effective with no risk of fistula formation. Prolonged postoperative bladder drainage is likely unnecessary, but more evidence is required by more reports of cases with similar outcomes. 
